# Pluripotent Stem Cell-Derived Gametes: Truth and (Potential) Consequences

**DOI:** 10.1016/j.stem.2009.06.005

**Published:** 2009-07-02

**Authors:** Debra J.H. Mathews, Peter J. Donovan, John Harris, Robin Lovell-Badge, Julian Savulescu, Ruth Faden

**Affiliations:** 1 Berman Institute of Bioethics, Johns Hopkins University, Baltimore, MD 21205, USA; 2 Sue and Bill Gross Stem Cell Research Center, University of California, Irvine, Irvine, CA 92697, USA; 3 Institute of Science, Ethics, and Innovation, University of Manchester, Manchester M13 9PL, UK; 4 Division of Stem Cell Biology and Developmental Genetics, MRC National Institute for Medical Research, London NW7 1AA, UK; 5 Oxford Uehiro Centre for Practical Ethics, University of Oxford, Oxford OX1 1PT, UK

## Abstract

An emerging body of data suggests that pluripotent stem cells may be able to differentiate to form eggs and sperm. We discuss the state of the science and the potential social implications and offer recommendations for addressing some of the ethical and policy issues that would be raised by the availability of stem cell-derived gametes.

## Main Text

Recent research suggests that it may be possible to derive gametes from a variety of pluripotent stem cell (PSC) sources (Clark et al., 2004, Geijsen et al., 2004, Hubner et al., 2003, Nayernia et al., 2006b, Park et al., 2009, Toyooka et al., 2003). Though preliminary, these findings may open doors to important discoveries in both basic and applied research. Furthermore, the science has already become a matter of debate and policy in at least two countries (Japan, 2000; United Kingdom, 2008a, 2008b). Significant hurdles face this science, as discussed below.

However, assuming that these obstacles can be overcome, the ability to create PSC-derived gametes raises a number of challenging ethical and policy issues that must be considered. To address such issues before they become pressing problems, we convened a group of more than 40 scientists, ethicists, journal editors, and lawyers to review and debate the challenges raised by PSC-derived gamete research. The objectives of this Hinxton Group (Hinxton Group, 2006) project included: (1) creating a road map for policymakers and the public, (2) providing relevant contextual information for applications related to PSC-derived gametes, and (3) providing guidance regarding ethical oversight. Here, we discuss the process and outcomes of the group's deliberations and our expansions on these deliberations in three parts: the state of the science, the societal implications, and recommendations.

### State of the Science

#### Current State of the Science

Though several scientists present at the meeting are directly involved in PSC-derived gamete research and therefore are likely to find it promising, others have no direct stake. Following considerable discussion, the group reached consensus that PSC-derived gamete research has “considerable scientific value and potential both for understanding basic mechanisms of gamete biology and overcoming clinical problems.” Thus far, scientists have been able to complete in vitro both the very early steps of gamete development from PSCs (e.g., Clark et al., 2004, Geijsen et al., 2004, Hubner et al., 2003, Nayernia et al., 2006a, Novak et al., 2006, Park et al., 2009, Toyooka et al., 2003) and later maturation steps of gametes that originated in vivo (e.g., Picton et al., 2008), but the intermediary steps bridging these two stages are proving more difficult (e.g., Novak et al., 2006). The latter is not surprising, as relatively little is known of germ cell biology during this period, such as erasure of genomic imprinting and cell-cycle control for entry into mitotic arrest or meiosis. Indeed, although one group has reported obtaining live born mice from PSC-derived male gametes, these pups died shortly after birth from defects likely to be related to imprinting problems (Nayernia et al., 2006b). Getting all the way from a human PSC to a gamete capable of fertilization—entirely in vitro—is likely to be years away. At the same time, the science is paying dividends right now in addressing questions about the role of specific genes in early germ cell development and the interaction between germ cells and supporting somatic cells.

#### Future of PSC-Derived Gametes

The most difficult issue that we faced in discussion about the science was predicting how fast this research would move and how long (if ever) it would be until PSC-derived gametes are used in clinical applications. The field of stem cell research has witnessed both major setbacks (Kennedy, 2006) and major leaps (Takahashi and Yamanaka, 2006) in recent years. Such unanticipated and profound events make already cautious and tentative scientists even more wary of forecasting timelines. However, we believe that derived human gametes are likely to be developed, but probably not for at least a decade. Clinical applications of derived gametes are unlikely to be available until several years after verifiable derived gametes can be reliably produced.

It will be critical to assess the quality of derived gametes. Observational and biochemical tests can measure some of their properties, but these assays are inadequate to judge whether the cells would support normal development. Determining functionality of derived gametes will, therefore, require establishing their capacity for fertilization and early embryogenesis. In other words, embryos will need to be created. One goal of this research, after all, is to generate sperm and eggs capable of making embryos and ultimately children. That said, the validity of research done entirely in vitro toward nonreproductive ends also will depend on this test of functionality if the in vitro findings are not to be subject to caveats about gamete quality.

Finally, critical to the policy discussion is what is not likely in the future of the science. That is, there has been discussion, in the press and in public and government deliberations, of the possibility of using PSC-derived gametes in same-sex reproduction. For example, if a gay female couple wanted to have a child that was genetically related to both partners, it has been said that this technology would enable sperm to be derived from one partner, which would then be used to fertilize the egg of the other partner using in vitro fertilization techniques. This scenario and its parallel in gay males, though headline grabbing, faces significant if not insurmountable scientific barriers. In brief, due to the complexity of the human egg and because it must contain all of the resources necessary to develop into an embryo, it will be very difficult to derive eggs that could be used for reproduction from XY (chromosomally male) cells, especially eggs able to give chromosomally normal offspring. The converse, deriving competent sperm from XX (chromosomally female) cells, faces so many scientific challenges—in particular, the fact that at least some of the genes critical for sperm formation are located on the Y chromosome—that it is difficult to envision how it would be possible given the current state of knowledge.

#### Utility of PSC-Derived Gametes

Research into PSC-derived gametes is already paying dividends—advancing our understanding of, for example, genes involved in early germline commitment and the interaction between germ cells and supporting cells. There are many reasons why basic scientists find the possibility of quantities of verified PSC-derived gametes attractive (see Table S1 available online). For example, having access to the earliest stages of developing gametes would allow scientists to study the factors affecting rates of chromosome nondisjunction during meiosis I (MI) and meiosis II (MII) in oocyte development, as well as the process of recombination, including hot spots and crossover formation. Although it is currently possible to obtain some human fetal oocytes from aborted fetuses to look at aspects of chromosome behavior and misbehavior during MI and to look at MII in oocytes donated for research by women after superovulation or from ovaries removed during hysterectomies, the numbers of oocytes that can be obtained in these ways is restricted, and for investigations of MI, each aborted fetus will be a different individual (and a different age), making it potentially difficult to compare one experiment with another.

Having only relatively small numbers of oocytes per experiment compromises certain types of investigations in which large numbers are needed—for example, to look for hot spots of recombination, as has been done for male meiosis; to carry out biochemistry on specific processes; or to screen chemicals, toxins, and pollutants for their effects on nondisjunction, etc. The ability to derive oocytes from human PSCs will, in theory, allow an unlimited number of these cells to be obtained at any stage of meiosis and at any stage of growth or maturation. SCNT or iPSC technology should allow oocytes to be derived from specific individuals carrying known (or unknown) mutations (including chromosome abnormalities), which will enable experiments exploring the effects of such mutations at stages that are otherwise inaccessible (e.g., MI).

Moreover, this set of assays can be done without compromising the individual's own fertility. For patients who may not have any oocytes left (e.g., premature ovarian failure), PSC-derived gametes may be the only way to study the mechanism responsible for oocyte loss.

A second example of basic science that is currently very challenging to conduct but would be made considerably more tractable with PSC-derived gametes are studies of the roles of specific genes in early gametogenesis. The ability to introduce genes or specific mutations into human PSCs will allow the role of these genes and alleles to be assessed in human germ cell development. Currently, this line of inquiry can be pursued in mice in vivo or in vitro, but it obviously cannot be undertaken in vivo in humans. Although some similarity between the two species is expected, functional parity cannot be taken for granted, as we know of specific differences already (e.g., in Y-linked genes required for spermatogenesis between humans and mice).

### Potential Social Implications

PSC-derived gamete research represents the convergence of several areas of ethical and policy debate and inquiry—stem cell research, human genetic research, reproductive technologies, and human enhancement—bringing many of today's most contentious ethical issues into the same conversation. Ethical and policy challenges are raised by both the potential applications of derived gamete research and by the science itself. The means and ends of this science will require deliberation by the public and policymakers to determine how these challenges should be managed, if at all (Table 1). As mentioned above, in order to verify that a given method of deriving human gametes from PSCs produces functional sperm and eggs, in vitro fertilization will need to be attempted, and any resulting embryos will need to be grown to at least the blastocyst stage. To be clear, derived gamete research will require the creation and destruction of human embryos; thus, this line of research will be morally objectionable to those who imbue human embryos with full moral status. In some countries, this procedure will also pose policy hurdles, particularly in jurisdictions where it is illegal to create human embryos exclusively for research purposes. Of note, this list includes a number of jurisdictions with permissive stem cell policies, which, for example, do not permit the fertilization of a human egg with a human sperm exclusively for research purposes but do permit somatic cell nuclear transfer.

However, even before PSC-derived gamete research reaches a stage at which human embryo creation and destruction becomes possible, these efforts are already producing and will continue to facilitate advances in basic science related to infertility and genetic disease, including chromosomal abnormalities and some cancers. Such advances may lead to treatments for these conditions. Following testing and validation of PSC-derived gametes, potential clinical applications include the creation of sperm and eggs for individuals who have lost their fertility due to disease, such as survivors of childhood cancers and women with premature ovarian failure and early menopause.

In addition, derived gamete research may ameliorate a current controversy in stem cell research. If scientists are able to generate functional human eggs from PSCs, the need to recruit women to provide their eggs via hormone-induced superovulation and retrieval and the risks and controversy inherent in that process may be eliminated. Until proven safe, PSC-derived eggs should be used only for research, whereas eggs from women undergoing hormone-induced superovulation could be reserved for fertility treatments. Ultimately, however, PSC-derived eggs may also eliminate controversies around soliciting women to make their eggs available for fertility treatment, whether by donation or with financial reward.

Though many of these applications are relatively noncontentious, some foreseen applications will clearly be controversial. For example, same-sex reproduction is inarguably a controversial, if highly unlikely, potential end result of this research. Germline genetic modification of humans, be it for the correction of disease mutations or genetic enhancement (for example, to confer disease resistance or increase height), will raise serious moral concerns for some. This technology may also facilitate the production of significantly larger quantities of eggs and, subsequently, embryos than current assisted reproductive technologies, vastly increasing the possibilities for embryo selection based on genetic profile. For example, if a couple is interested in selecting embryos for implantation based on multiple alleles, whether related to disease risk or phenotypic traits such as eye color, the potential mother's PSC-derived eggs could be used to create hundreds of embryos, ensuring that all of the desired alleles are present together in at least one embryo. This approach will not only raise concerns about the creation of huge numbers of embryos in excess of clinical need, but will also fuel debates about designer babies and which traits, if any, are legitimate targets of selection.

The convergence of PSC-derived gamete technology with other new technologies, notably induced pluripotent stem cells (iPSCs), may force societies to confront additional challenging situations, including the creation of embryos from the tissues of fetuses, children, or the deceased. If scientists are able to generate gametes from somatic tissues, all three of these scenarios will become possible and practical.

Finally, the ability to generate large numbers of human gametes (with random or designed genetic constitutions) will enable the practice of in vitro human genetics. That is, scientists will be able to conduct multigenerational human genetic studies in a dish, for example, to track the impact of various environmental conditions on the development of human disease or the impact of crossing specific genotypes. Such research may also facilitate the generation of ideal “universal donor” cells, with appropriate combinations of haplotypes at histocompatibility loci.

Although many individuals will welcome the prospects for disease prevention and health promotion that such research should facilitate, many others will find the treatment of human embryos in such blatantly instrumental ways to be ethically unacceptable. We highlight these examples to bring into sharp relief a constitutive feature of this and many other emerging technologies: the science will facilitate both “acceptable” and “unacceptable” means and ends. Determining which means and ends fall into which category will be up to individuals and societies. Further, societies will need to determine how they will deal with the dual use nature of this research.

### Recommendations

Societies will respond differently to the charge of how to regulate and enforce policies designed to oversee the conduct of PSC-derived gamete research. It is important to note that many of our recommendations (Table 2) apply only to jurisdictions in which the practice of this area of science is legal. In Japan, for example, where deriving gametes from PSCs is illegal (Japan, 2000), much of what we have to say will not currently apply, although ramifications of the research may need to be accommodated over time, for example, if “medical tourism” follows any successful use of PSC-derived gametes for infertility treatment. Some of these issues may be addressed in existing recommendations (e.g., International Society for Stem Cell Research, 2006, 2008), but in jurisdictions where the research itself is permitted, scientists, the public, and policymakers will need to consider a variety of issues as the science progresses.

Currently, many jurisdictions and research institutions require specific consent from tissue donors for the use of their tissues (e.g., sperm and supernumerary IVF embryos) in stem cell research. A similar requirement should exist for tissue donors whose cells are used to derive gametes that are intended for use in reproduction, just as those whose native gametes are used in reproduction must give explicit consent. This obligation is meant to include those donors whose skin or other somatic tissues are manipulated to become gametes for reproduction through iPSC technology. This recommendation automatically rules out the reproductive use of embryos involving gametes derived from tissue sources from whom valid informed consent cannot be obtained, including fetuses, minors, and the deceased (with the possible exception of written consent prior to death). That said, we do not believe that specific consent must be required of tissue donors for the use of their tissues to derive gametes through iPSC technology that are intended for in vitro use only, without the production of embryos.

In addition, prior to the initial use of these cells for reproductive purposes, appropriate oversight structures must be in place. In vitro fertilization (IVF) and intracytoplasmic sperm injection (ICSI) were first undertaken in humans with very little oversight (Cohen et al., 2005, Devroey and Van Steirteghem, 2004; Warnock Report, 1984), and even today, there is scant systematic data about the effects of these interventions on women and children (Allen et al., 2006). The early use of derived gametes in human reproduction should follow a different path. First, early attempts should take place only within the context of carefully conducted clinical research that conforms to the highest ethical standards. Second, the health and well-being of female participants and their developing fetuses should be monitored carefully. Pregnancy outcomes should be recorded, and the health and well-being of children born should be monitored in long-term follow-up studies.

Though social values should be part of any policy discussion that takes place, policymakers should not attempt to restrict scientific inquiry solely because there are divergent moral views among interested parties. That is, the mere presence of moral disagreement does not justify the regulation of the science; as science progresses, moral disagreement is inevitable. Moral disagreement does, however, signal the need for public discussion and debate and the engagement of scientists with the public and policymakers. Some disagreements about the methods or consequences of science reflect deep-seated differences in moral standpoints that are not easily reconcilable and that will require policymakers to take substantive positions that will remain unacceptable to some. Insofar as the result of such a process is the development of a restrictive policy, it is important to target it specifically to those dimensions of the research or its applications that have been determined to be unacceptable. It is also important that these policies be proportionate to the magnitude of what is morally at stake.

### Conclusion

In 2006, we called for scientists to be vigilant in forecasting coming ethical challenges and to engage in efforts to address the ethical issues before they could become concrete problems (Mathews et al., 2006). We further called for the development of strategies to foster the conduct of scientifically and ethically defensible research. This project was an attempt to do both. PSC-derived gamete research does and will raise a variety of ethical and policy challenges, yet the public debate has not yet begun in most countries. Societies need to begin discussing the issues raised by derived gamete research and its potential applications and doing the work necessary to determine the best policy response to the risks, benefits, and potential moral concerns involved.

## Figures and Tables

**Table 1:** Issues Requiring Deliberation and Possible Policy Options

Issues	Policy Options				
Use of PSCDGs in Research	prohibited	restricted	permitted	funded	
Creation of Embryos from PSCDGs	prohibited	research only	research and reproduction		
Access to PSCDG-Based Reproduction	restricted	equivalent to IVF availability	same-sex couples	postmenopausal women	without informed consent (e.g., minors)

**Table 2:** Recommendations

1. Policymakers should not attempt to restrict scientific inquiry solely because there are divergent moral views among interested parties.
2. Restrictive policies should be targeted to those dimensions of the research or its applications that have been determined to be unacceptable and should be proportionate to the magnitude of what is morally at stake.
3. Specific consent need not be required of tissue donors for the use of their tissues to derive gametes that are intended for in vitro use only, without the production of embryos.
4. Prior to reproductive uses of these cells commencing, appropriate oversight structures must be in place.
5. There should be requirements for specific consent by tissue donors for use of their tissues to derive gametes that are intended for use in reproduction. This is meant to include those whose skin or other somatic tissues are manipulated to become gametes through iPSC technology. This would also rule out the use of tissue from fetuses, minors, and the deceased for these purposes.
6. Early attempts should take place only within the context of carefully conducted clinical research that conforms to the highest ethical standards.
7. The health and well-being of female participants and their developing fetuses should be monitored carefully. Pregnancy outcomes should be recorded. The health and well-being of children born should be monitored in long-term follow-up studies.

## Web Resources

Hinxton Group. (2006). http://www.hinxtongroup.org.
International Society for Stem Cell Research. (2006). Guidelines for the conduct of human embryonic stem cell research. http://www.isscr.org/guidelines/ISSCRhESCguidelines2006.pdf.
International Society for Stem Cell Research. (2008). Guidelines for the clinical translation of stem cells. http://www.isscr.org/clinical_trans/pdfs/ISSCRGLClinicalTrans.pdf.
Japan. (2000). The guidelines for derivation and utilization of human embryonic stem cells. http://www.lifescience.mext.go.jp/files/pdf/32_90.pdf.
United Kingdom. (2008a). The Lords Hansard, 3 Dec 2007: Column 1496. http://www.parliament.the-stationery-office.co.uk/pa/ld200708/ldhansrd/text/71203-0002.htm.
United Kingdom. (2008b). Human Fertilisation and Embryology Act 1990 - an illustrative text. http://www.dh.gov.uk/en/Publicationsandstatistics/Publications/PublicationsLegislation/DH_080205.
Warnock Report. (1984). Report of the Committee of Inquiry into Human Fertilisation and Embryology. http://www.hfea.gov.uk/docs/Warnock_Report_of_the_Committee_of_Inquiry_into_Human_Fertilisation_and_Embryology_1984.pdf.

## Supplemental Data

Document S1. One Table
