# Supplementary material for: Pluripotent Stem Cell-Derived Gametes: Truth and (Potential) Consequences
Source: Cell Stem Cell. 2009 Jul 2;5(1):11–4. doi: 10.1016/j.stem.2009.06.005 (PMC5226919; doi:10.1016/j.stem.2009.06.005)
Supplement: Document S1. One Table [file mmc1.pdf]

Cell Stem Cell, ***Volume 5***

Supplemental Data

Pluripotent Stem Cell-Derived Gametes: Truth and (Potential) Consequences

Debra J.H. Mathews, Peter J. Donovan, John Harris, Robin Lovell-Badge, Julian Savulescu, and Ruth Faden

Table S1. Scientific Potential

	Application	New scientific possibilities with PSCDG	Scientific possibilities greatly facilitated by PSCDG
**Basic Science Applications**	Gametes derived from pluripotent stem cells for use in research	1) Gene programming in PSCDGs;<br>2) Differences in programming/gene expression between in vivo derived cells and PSCDGs;<br>3) Study of genetic diseases that affect germ cell biology and are caused by genes not well conserved from mouse to human, such as those on the X and Y chromosomes;<br>4) A tool for gene knock-out studies in human germ cells and gametes;<br>5) Increased supply of oocytes enabling proteomic studies, drug screening, etc.	1) Source of oocytes for use in SCNT;<br>2) Early specification of primordial germ cells;<br>3) Mechanisms that erase and re-establish genomic imprinting;<br>4) X-chromosome re-activation;<br>5) Entry into mitotic arrest, which is characteristic of germ cells in the fetal testis;<br>6) Entry into meiosis, which is characteristic of germ cells in the fetal ovary;<br>7) *°Factors affecting rates of chromosome non-disjunction during meiosis I and or meiosis II in oocyte development;<br>8) Meiotic chromatin organization;<br>9) *°Recombination, including hotspots, crossover formation;<br>10) The formation of follicles and control of oocyte growth and maturation;<br>11) Rates of oocyte growth and maturation - determined intrinsically or extrinsically?;<br>12) Mitochondrial inheritance, genetics and function;<br>13) Centrosome inheritance and function;<br>14) Factors that trigger and regulate spermatogenesis;<br>15) Flagellar function;<br>16) Spermiogenesis and acrosome function;<br>17) Influence of the somatic cell environment and of extrinsic factors such as hormones on all of the above;<br>18) *Role played by specific genes in these processes;<br>19) Diseases with origins in meiosis;<br>20) Germ cell tumor origination;<br>21) Transgenerational epigenetic inheritance;<br>22) Toxicology-related effects on germ cell development
	PSCDG to create embryos for research	1) In vitro genetics	1) Centrosome inheritance and function;<br>2) Mitochondrial inheritance, genetics and function;<br>3) Mechanisms that erase and re-establish genomic imprinting;<br>4) X-Chromosome reactivation;<br>5) Gonad formation;<br>6) Diseases related to imprinting;<br>7) Identify regions of the genome that tolerate insertions, for eventual genetic modification
	Germline genetic modification and PSCDG as a research tool	1) Research to advance human germline genetic modification for reproductive use	1) *Specific genes and pathways;<br>2) Genetic aspects of disease;<br>3) Disease etiology
**Clinical Applications**	Increased reproductive genetic screening using PSCDG	1) Develop methods for screening oocytes either for quality or for desired traits;<br>2) Cell biology of gametes from those who are infertile	1) Genetic screening of embryos for desired traits;<br>2) Develop methods for screening sperm either for quality or desired traits
	PSCDG to create gametes for use with ART/IVF	1) Genetically-related children for those who have lost fertility due to disease (including infectious diseases and cancer) or its treatment (such as radiotherapy, chemotherapy, or gonadectomy), etc.;<br>2) Genetically-related children for women with premature ovarian failure, which can be due to genetic defects affecting the eggs or their supporting follicle cells, or to hormonal defects;<br>3) Genetically-related children for post-menopausal women;	1) Bypass problems limiting the production of viable gametes for individuals to use with IVF techniques

		4) Genetically-related children for men or women with sex reversal syndromes or individuals with certain intersex conditions;<br>5) Genetically-related children of same-sex parents;<br>6) Children from a single donor	
	PSCDG to modify the human germline for genetic correction of disease traits	1) Pluripotent stem cells can be genetically manipulated in culture; Successfully modified pluripotent stem cells can then be used to derive gametes genetically modified to correct disease traits	
	PSCDG to modify the human germline for enhancement/diversity	1) Pluripotent stem cells can be genetically manipulated in culture; Successfully modified pluripotent stem cells can then be used to derive gametes genetically modified for enhancement	

***** Research that is *particularly* challenging without PSCDGs; °Oocyte development begins in the fetal stage, with much of MI (in particular, recombination) occurring before birth. At birth, a human female already has her full complement of oocytes, and they are suspended in MI; meiosis does not resume until ovulation. Non-disjunction during MI is a primary cause of chromosome abnormality in early human embryos, and consequently is responsible for much early embryonic loss.
